# Supplementary material for: Synaptic disruption and CREB‐regulated transcription are restored by K+ channel blockers in ALS
Source: EMBO Mol Med. 2021 Jun 14;13(7):e13131. doi: 10.15252/emmm.202013131 (PMC8261490; doi:10.15252/emmm.202013131)
Supplement: Supplementary file 1 — Appendix [file EMMM-13-e13131-s003.pdf]

Synaptic disruption and CREB-regulated transcription are restored by K^+ channel blockers in ALS

Alberto Catanese[1,‡], Sandeep Rajkumar[1], Daniel Sommer[1], Dennis Freisem[1], Alexander Wirth[1], Amr Aly[1], David Massa-López[2], Andrea Olivieri[1], Federica Torelli[1], Valentin Ioannidis[1], Joanna Lipecka[3], Ida Chiara Guerrera[3], Daniel Zytnicki[4], Albert Ludolph[2,5], Edor Kabashi[6], Medhanie A Mulaw[7], Francesco Roselli[1,2,5, ‡], Tobias M Böckers[1,2, ‡]

Affiliations:

[1] Institute of Anatomy and Cell Biology, Ulm University School of Medicine, Ulm, Germany

[2] Deutsches Zentrum für Neurodegenerative Erkrankungen (DZNE), Ulm site, Ulm, Germany

[3] Proteomics platform Necker, Université de Paris - Structure Fédérative de Recherche Necker, INSERM US24/CNRS UMS3633, Paris 75015, France.

[4] Université de Paris, SPPIN - Saints-Pères Paris Institute for the Neurosciences, CNRS, Paris

[5] Dept. of Neurology, Ulm University School of Medicine, Ulm, Germany

[6] Institute of Translational Research for Neurological Disorders, INSERM UMR 1163, Imagine Institute, Paris, France

[7] Internal Medicine I and Institute of Molecular Medicine and Stem Cell Aging, Medical Faculty, University Hospital Ulm and University of Ulm University, Ulm, Germany

[‡] Co-senior author

Appendix

Table of contents:

Appendix Figure S1

Appendix Figure S2

Appendix Figure S3

Appendix Figure S4

Appendix Figure S5

Appendix Figure S6

Appendix Figure S7

Appendix Figure S8

Appendix Figure S9

Appendix Figure S10

Appendix Figure S11

Appendix Figure S12

Appendix Table S1

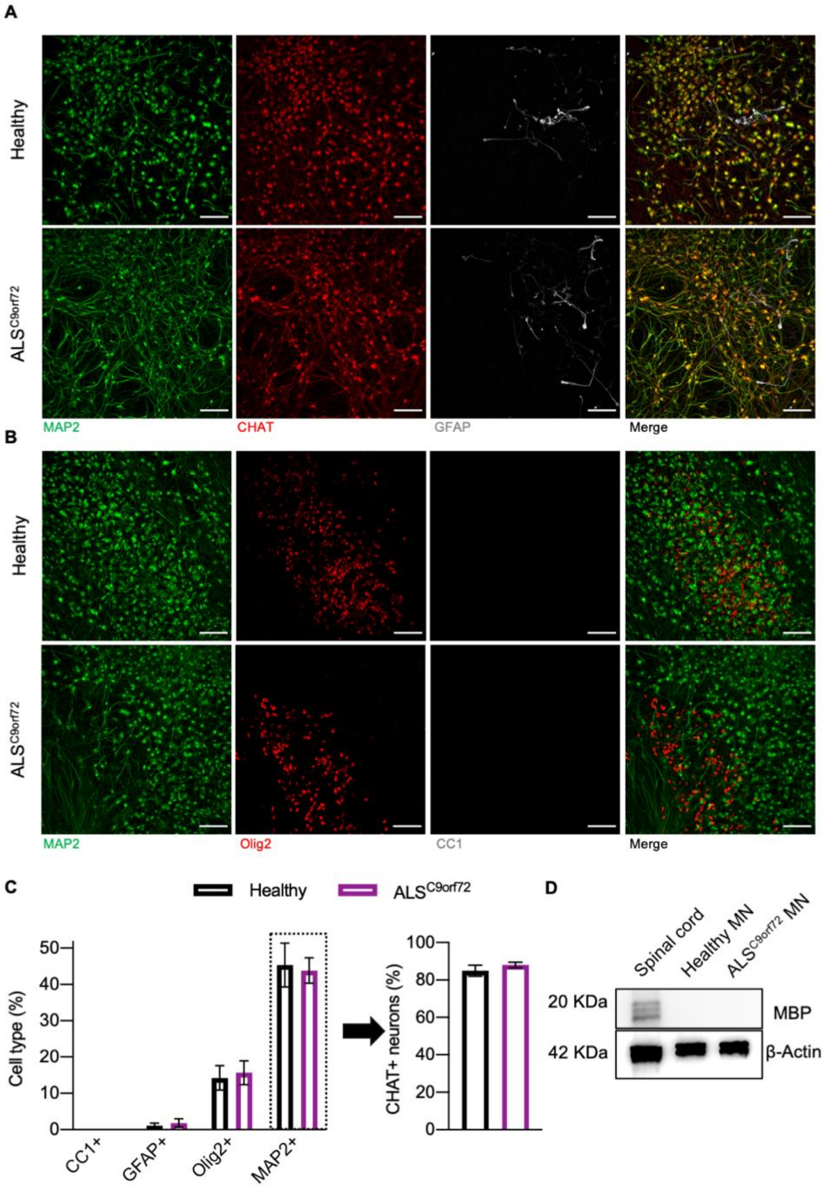

Appendix Figure S1. Characterization of the hiPSC-derived MN cultures.

(A) Representative images of DIV21 Healthy and ALS^{C9orf72} hiPSC-derived MN stained against MAP2, CHAT, and the astrocyte marker GFAP. Scale bars: 100 µm.

(B) Representative images of DIV21 Healthy and ALS^{C9orf72} hiPSC-derived MN stained against MAP2, and the MN precursor marker Olig2. The absence of cells positively stained against CC1 indicates the absence of oligodendrocytes in cultures. Scale bars: 100 µm.

(C) Quantification of the different cell types identified in the hiPSC-derived MN cultures. No differences between Healthy and ALS^{C9orf72} genotypes were found (Welch´s t-test). For each genotype, n=3 independent differentiations from two hiPSC lines were analyzed. Healthy I and Healthy II were used as representative of the controls, while ALS^{C9orf72} II and ALS^{C9orf72} III as representative of the mutants.

(D) Western blot showing the presence of the oligodendrocyte marker MBP in the total lysate of murine spinal cord, and its absence in lysates from Healthy and ALS^{C9orf72} hiPSC-derived cultures.

Data information: error bars represent SEM.

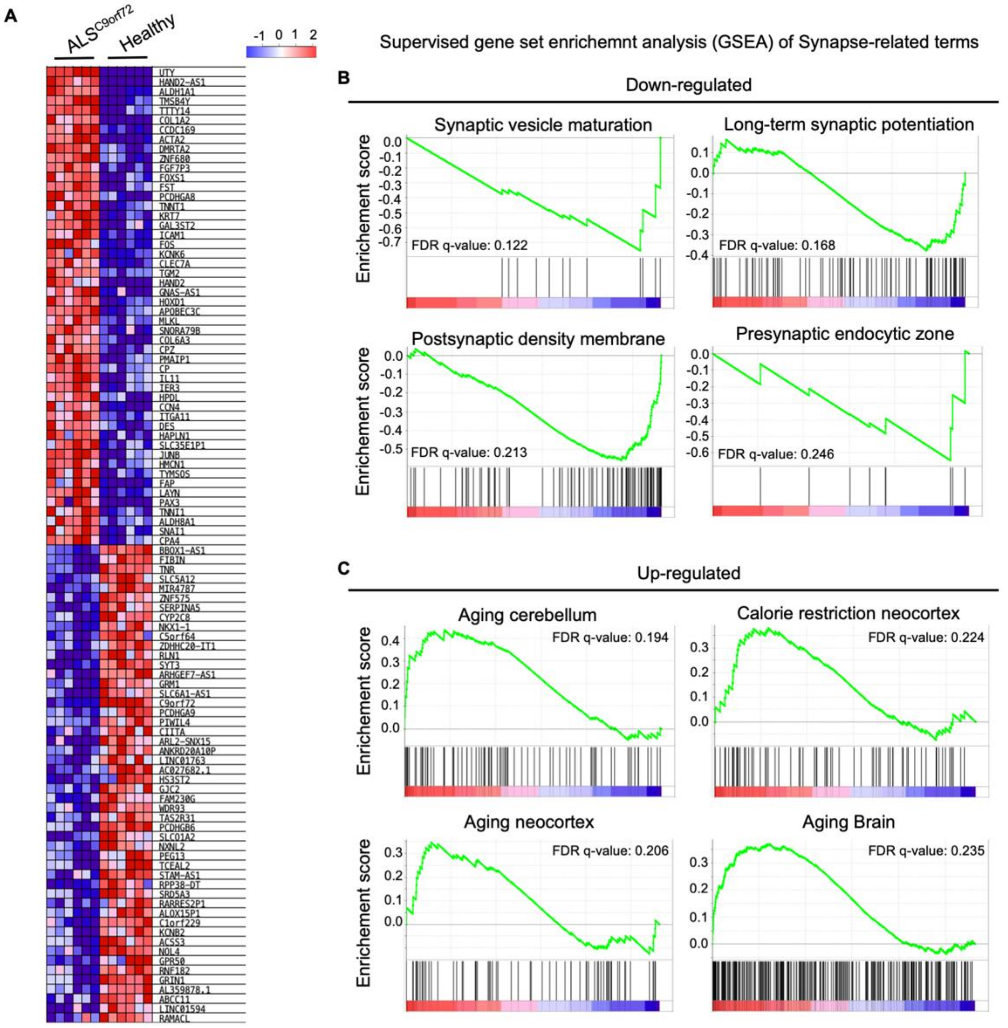

Appendix Figure S2. Supervised GSEA confirms synaptic alterations in the transcriptomes of ALS^{C9orf72} MN.

(A) Heatmap representing the top 50 up- and down-regulated genes in mutant MN identified with GSEA.

(B) Enrichment plots of representative down-regulated terms in ALS^{C9orf72} MN.

(C) Enrichment plots of representative up-regulated terms in ALS^{C9orf72} MN.

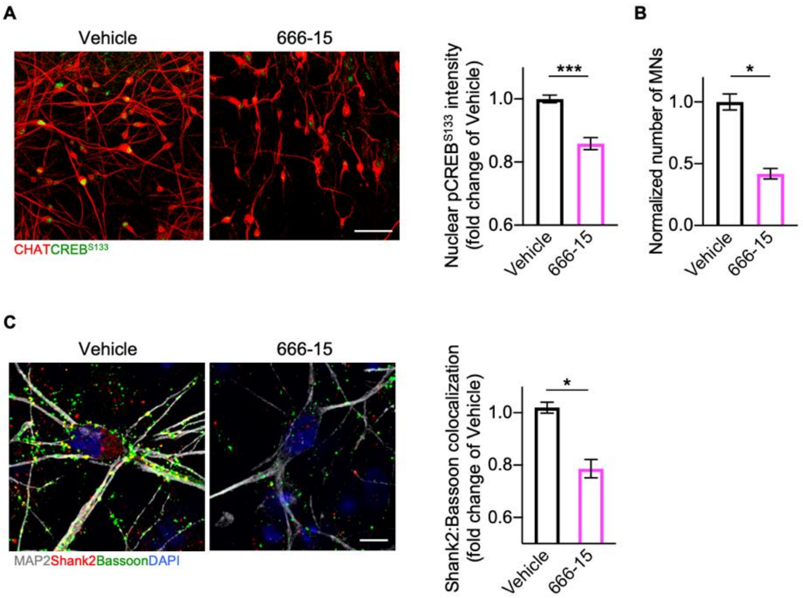

Appendix Figure S3. Pharmacological inhibition of CREB triggers MN and synapse loss in Healthy cultures.

(A) Treatment of Healthy I MN with CREB-inhibitor 666-15 significantly reduces the levels of pCREB^{S133} (Mann-Whitney test). n= 3 independent treatments performed with the Healthy I line. Scale bar: 50 µm.

(B) After 24 hours of treatment, CREB inhibition induces loss of Healthy MN (Welch´s t-test).

(C) Upon 666-15 treatment, the number of excitatory synapses is significantly reduced as well in comparison to vehicle-treated cultures (Welch´s t-test). n= 3 independent treatments performed with the Healthy I line. Scale bar: 10 µm.

Data information: *p<0.05; ***p<0.001. Error bars represent SEM. Exact p-values are reported in Appendix Table S1.

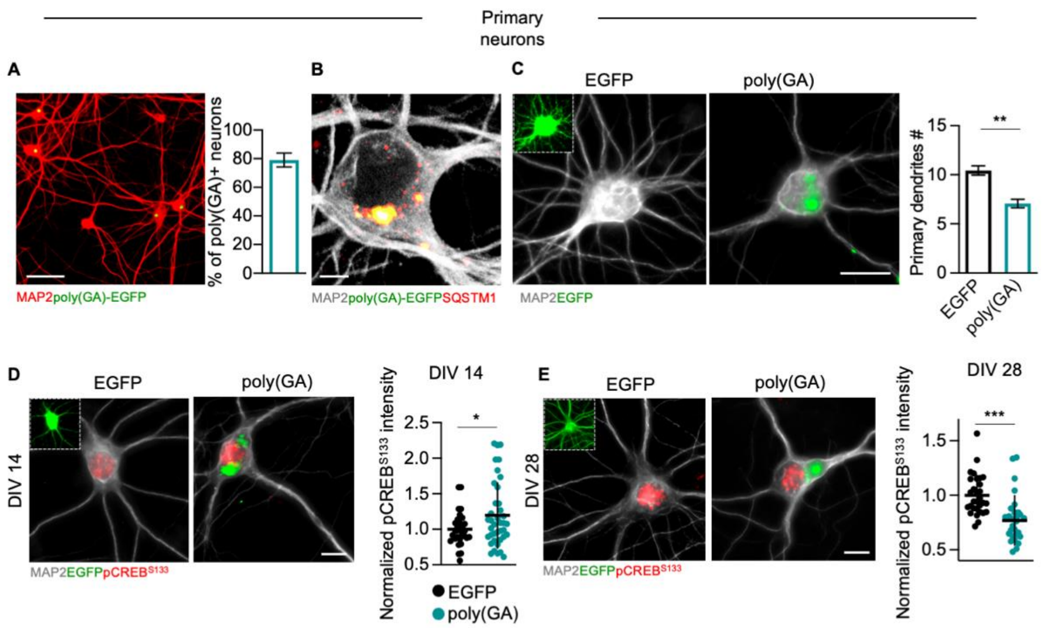

Appendix Figure S4. Overexpression of poly(GA) in primary cortical neurons recapitulates alterations similar to those observed in human C9orf72-mutant MN.

- **(A)** 78% of primary neurons are efficiently transduced by the AAV9-hSyn-poly(GA)$_{175}$-EGFP vector. n=3 independent cultures. Scale bar: 30 µm.
- **(B)** Poly(GA) aggregates sequestrate the autophagy receptor SQSTM1/p62. Scale bar: 5 µm.
- **(C)** Overexpression of poly(GA) reduces the number of primary dendrites in cortical cultures (Mann-Whitney test). n=3 independent cultures. Scale bar: 15 µm.
- **(D)** At DIV 14, poly(GA)+ cortical neurons have significantly higher levels of nuclear pCREB^{S133} than EGFP-positive cells (Welch´s t-test). n=32 neurons from 3 independent cultures. Scale bar: 15 µm.
- **(E)** The nuclear intensity of pCREB^{S133} becomes significantly lower in poly(GA)+ neurons than in the EGFP control cultures at DIV 28 (Mann-Whitney test). n=30 neurons from 3 independent cultures. Scale bar: 15 µm.

Data information: *p<0.05; **p<0.01. Error bars represent SEM. Exact p-values are reported in Appendix Table S1.

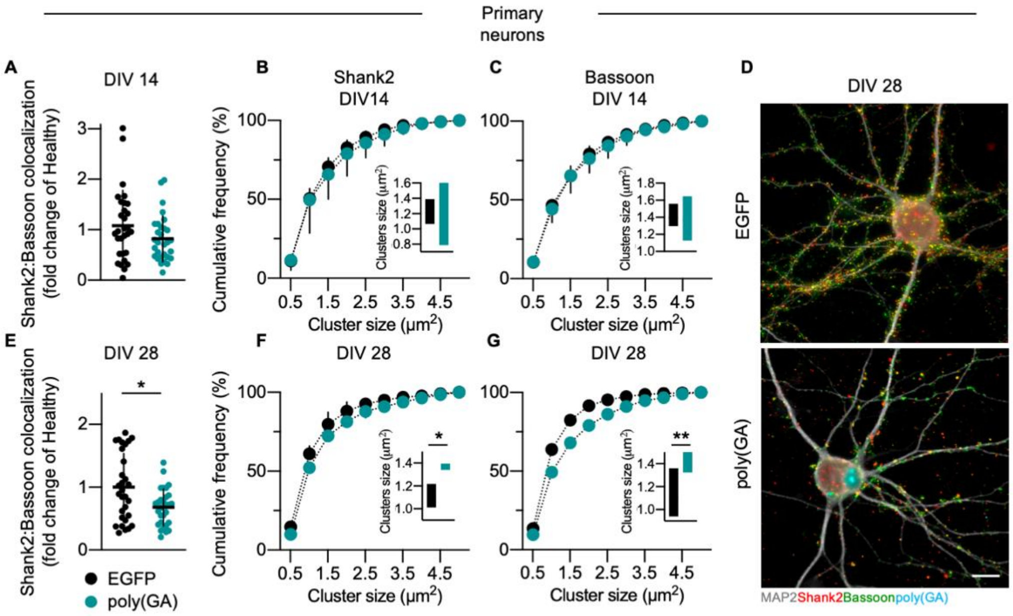

Appendix Figure S5. Time-dependent loss of excitatory synapses occurs upon poly(GA) overexpression.

- **(A)** At DIV 14, poly(GA) overexpression does not induce synapse loss (Mann-Whitney test). n=30 neurons from 3 independent cultures.
- **(B)** The size of Shank2 synaptic clusters is comparable between DIV 14 EGFP and poly(GA)+ neurons (Welch´s t-test).
- **(C)** The size of Bassoon synaptic puncta is not altered by poly(GA)+ accumulation at DIV 14 as well (Welch´s t-test).
- **(D)** Representative picture of DIV 28 neurons showing a loss of synaptic contacts upon poly(GA) overexpression. Scale bar: 10 µm.
- **(E)** The number of Shank2:Bassoon colocalizing spots is significantly reduced in DIV 28 poly(GA)+ neurons than in those expressing EFGP (Mann-Whitney test). n=30 neurons from 3 independent cultures.
- **(F)** DIV 28 neurons transduced with poly(GA) show aberrant accumulation of dendritic Shank2 puncta (Welch´s t-test).
- **(G)** The size of Bassoon clusters of DIV 28 poly(GA)+ neurons is also significantly larger than those detected in EGFP+ cortical cells (Welch´s t-test).

Data information: *p<0.05; **p<0.01. Error bars represent SEM. Exact p-values are reported in Appendix Table S1.

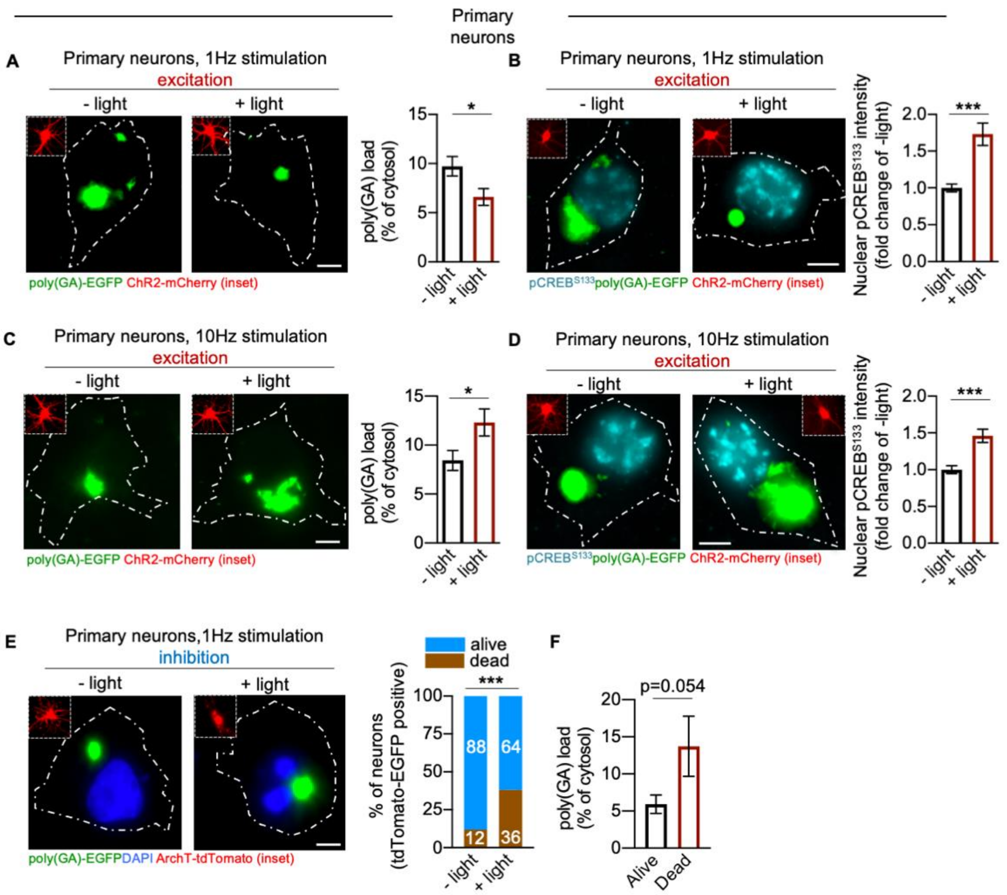

Appendix Figure S6. Optogenetic manipulation of neuronal activity impacts poly(GA) accumulation and neuronal survival in primary cortical cultures.

- **(A)** Low frequency (1Hz) optogenetic stimulation of poly(GA):ChR2+ primary neurons reduces the accumulation of poly(GA) aggregates (Mann-Whitney test). n=30 cells from 3 independent treatments. Scale bar: 5µm.
- **(B)** 1Hz stimulation significantly increases the levels of nuclear pCREB^{S133}. n=30 cells from 3 independent treatments (Welch´s t-test). Scale bar: 5µm.
- **(C)** High frequency (10Hz) stimulation strongly enhances the accumulation of poly(GA) aggregates, suggesting a frequency-dependent beneficial effect of increased neuronal firing. n=30 cells from 3 independent treatments (Welch´s t-test). Scale bar: 5µm.
- **(D)** 10Hz stimulation also significantly increases the levels of nuclear pCREB^{S133} (Mann-Whitney test). n=30 cells from 3 independent treatments. Scale bar: 5µm.
- **(E)** Neuronal inhibition at 1Hz increases the number of apoptotic neurons in poly(GA):Arch+ cortical neurons (Fisher's exact test). n=30 cells from 3 independent treatments. Scale bar: 5µm.
- **(F)** The load of poly(GA) aggregates in apoptotic ArchT-tdTomato+ neurons is higher than in surviving neurons upon light stimulation (Mann-Whitney test). n=30 cells from 3 independent treatments.

Data information: *$p<0.05$; ***$p<0.001$. Error bars represent SEM. Exact p-values are reported in Appendix Table S1.

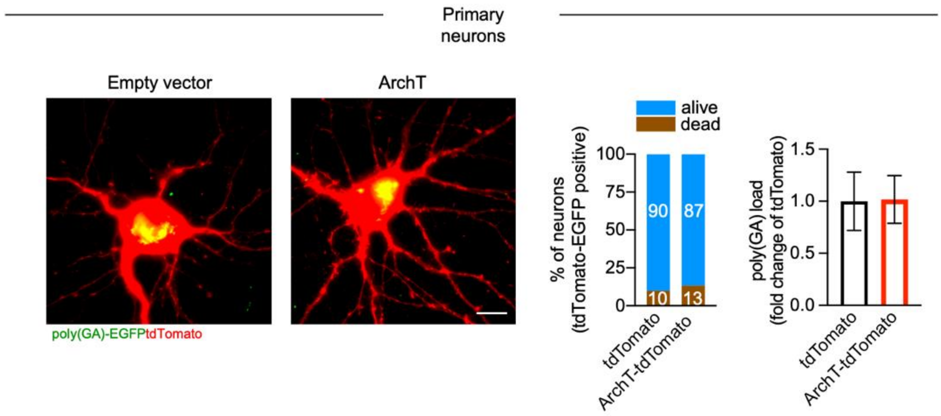

Appendix Figure S7. Expression of ArchT-tdTomato has no effect on the neuronal viability and aggregate accumulation of poly(GA)-expressing primary cells.

Transfection with the inhibitory opsin does not induce any detrimental effect in poly(GA)+ cortical neurons in absence of light stimulation (Fisher's exact test and Welch´s t-test). n=30 neurons from 3 independent treatments. Scale bar: 10µm.

Data information: error bars represent SEM.

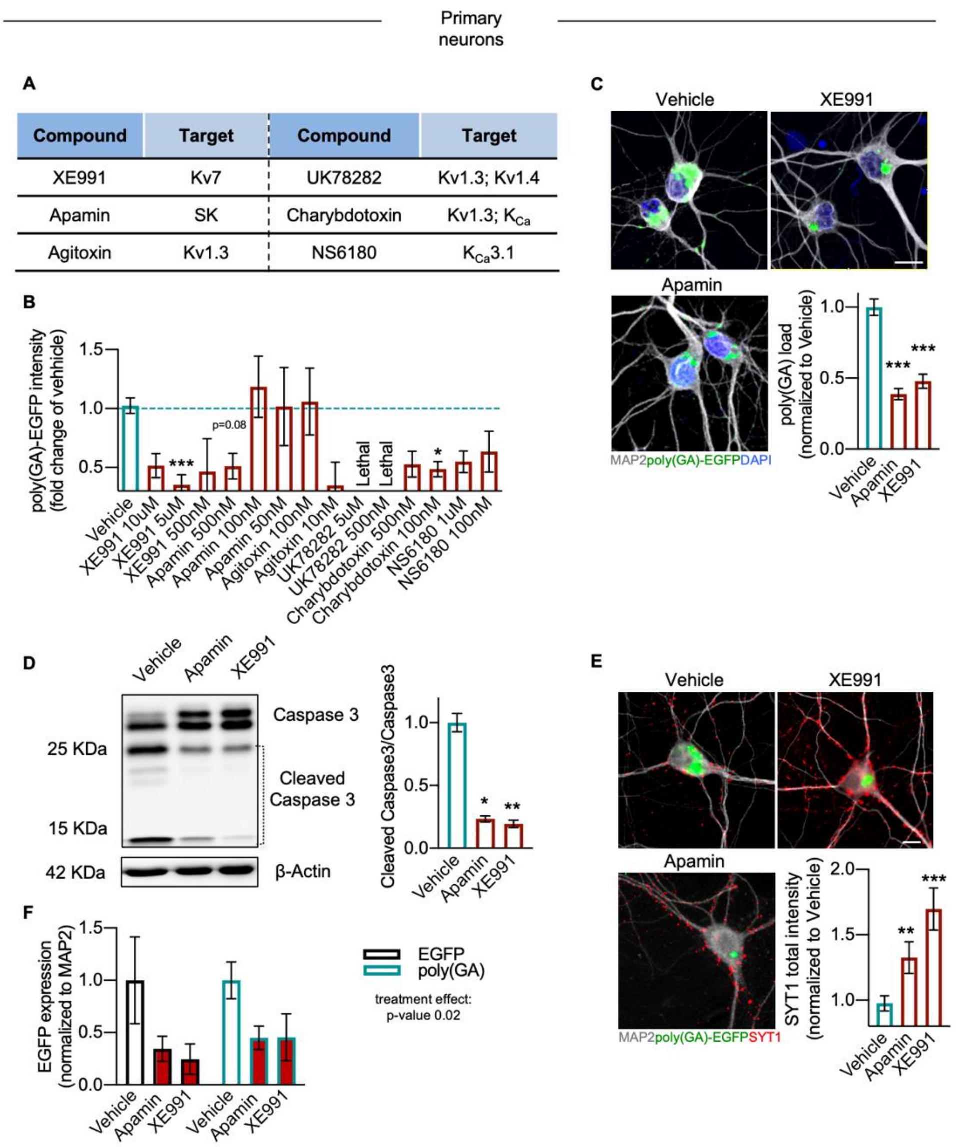

Appendix Figure S8. Neuroprotective effect of Apamin and XE991 in poly(GA)-expressing primary neurons.

- **(A)** Summary table displaying the different K^+ channel blockers (and their targets) tested.
- **(B)** Analysis of the EGFP intensity measured in poly(GA)+ neurons treated with K^+ channel blockers (Kruskal-Wallis test). (Kruskal-Wallis test). n≥7 wells for each treatment analyzed with a multiwell plate reader.
- **(C)** Confirmation of the beneficial effect of Apamin and XE991 treatment in reducing poly(GA) accumulation. n=3 independent treatments (one-way ANOVA followed by Dunnett's multiple comparison test). Scale bar: 10µm.
- **(D)** Both K^+ channel blockers reduce the levels of cleaved caspase 3 in primary neurons expressing poly(GA) (one-way ANOVA followed by Dunnett's multiple comparison test). n=3 independent treatments.
- **(E)** K^+ channel blockade increases the intensity of SYT1+ dendritic puncta, indicating increased synaptic activity. n=3 independent treatments (one-way ANOVA followed by Dunnett's multiple comparison test). Scale bar: 10µm.
- **(F)** Treatment with Apamin and XE991 reduces the mRNA levels of EGFP and poly(GA)-EGFP, suggesting increased degradation of these transcripts (two-way ANOVA). n=3 independent treatments.

Data information: *p<0.05; **p<0.01; ***p<0.001. Error bars represent SEM. Exact p-values are reported in Appendix Table S1.

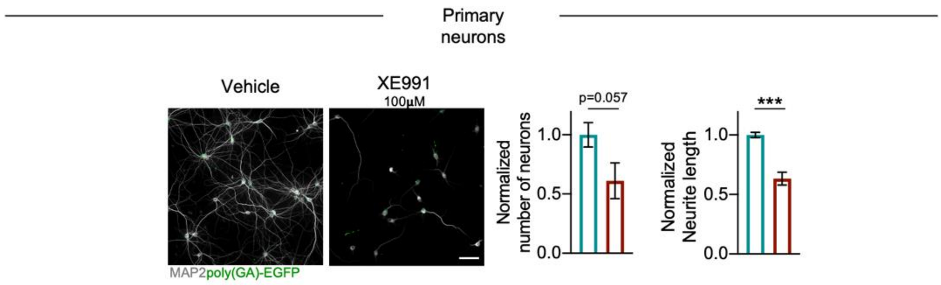

Appendix Figure S9. Higher dose of XE991 induces neuronal loss in poly(GA)+ cultures.
XE991 100µM significantly reduces neuronal survival and the neurite length of poly(GA)+ neurons (Welch´s t-test). n=3 independent treatments. Scale bar: 50µm.
Data information: ***p<0.001. Error bars represent SEM. Exact p-values are reported in Appendix Table S1.

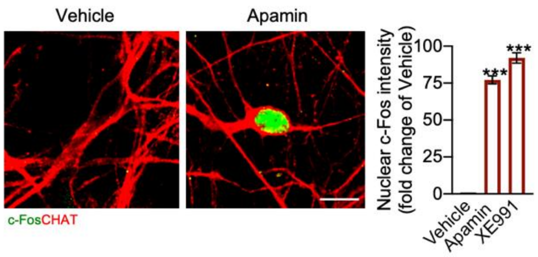

Appendix Figure S10. Apamin and XE991 increase the levels of the immediate early gene c-Fos, indicating increased MN activity.

ALS^{C9orf72} II MN treated with both K^+ channel blockers are characterized by significantly higher levels of nuclear c-Fos than vehicle-treated cultures (Kruskal-Wallis test). n=3 independent treatments. Scale bar: 10 µm.

Data information: ***p<0.001. Error bars represent SEM. Exact p-values are reported in Appendix Table S1.

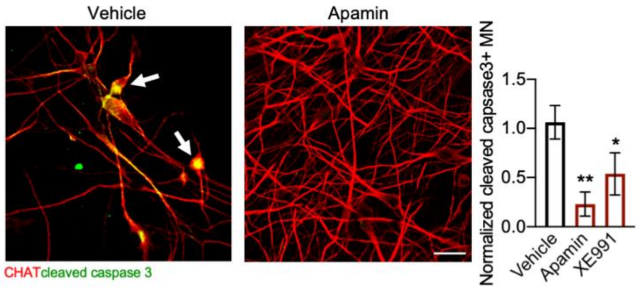

Appendix Figure S11. Apamin and XE991 reduce MN apoptosis in ALS^{C9orf72} cultures.
Blockade of K^+ channels significantly reduces the number of cleaved caspase 3-positive MN in mutant cultures (one-way ANOVA followed by Dunnett's multiple comparison test). n=3 independent treatments performed with the ALS^{C9orf72} I line. Scale bar: 30 µm.
Data information: *p<0.05; **p<0.01. Error bars represent SEM. White arrows indicate cleaved caspase 3+ MN. Exact p-values are reported in Appendix Table S1.

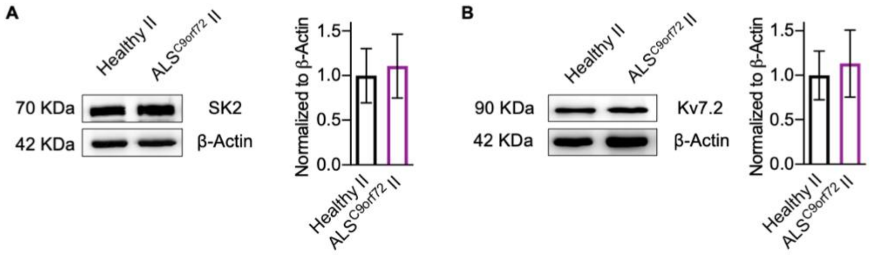

Appendix Figure S12. Expression of SK2 and Kv7.2 channels is not altered in ALS^{C9orf72} MN.

(A) Representative western blot showing no differences between Healthy and ALS^{C9orf72} MN in the levels of SK2 channel, target of Apamin (Welch´s t-test). n=3 independent cultures with the Healthy II and ALS^{C9orf72} II lines as representative of the corresponding genotype.

(B) The levels of the Kv7.2 channel, target of XE991, are comparable between cultures belonging to the different genotypes as well (Mann-Whitney test). n=3 independent cultures with the Healthy II and ALS^{C9orf72} II lines as representative of the corresponding genotype.

Data information: error bars represent SEM.

Figure	Panel (comparison)	Statistical test	p-value
1	F (NLGN3)	Welch`s t test	0.0026
	F (SLC6A)	Welch`s t test	0.0110
	F (TRIM9)	Welch`s t test	0.0469
	F (SYT1)	Welch`s t test	0.0237
	F (SYNGR1)	Welch`s t test	0.0455
	F (SNAP91)	Welch`s t test	0.0685
	G (Healthy *vs* $ALS^{C9orf72}$, DIV 21)	Two-way ANOVA	<0.001
	G (Healthy *vs* $ALS^{C9orf72}$, DIV 58)	Two-way ANOVA	0.0120
	G (Healthy *vs* $ALS^{C9orf72}$, DIV 70)	Two-way ANOVA	0.0010
	H (Healthy *vs* $ALS^{C9orf72}$, DIV 58)	Two-way ANOVA	0.0208
	H (Healthy *vs* $ALS^{C9orf72}$, DIV 70)	Two-way ANOVA	0.0003
2	A (Vehicle *vs* Apamin)	One-way ANOVA	<0.001
	A (Vehicle *vs* XE991)	One-way ANOVA	<0.001
	C (Vehicle *vs* Apamin)	Kruskal-Wallis test	<0.001
	C (Vehicle *vs* XE991)	Kruskal-Wallis test	<0.001
	D (Vehicle *vs* Apamin)	One-way ANOVA	0.0307
	D (Vehicle *vs* XE991)	One-way ANOVA	0.0154
	E (Vehicle *vs* Apamin)	One-way ANOVA	0.0117
	E (Vehicle *vs* XE991)	One-way ANOVA	0.0022
	F (Vehicle *vs* Apamin)	One-way ANOVA	<0.001
	F (Vehicle *vs* XE991)	One-way ANOVA	<0.001
3	E (Vehicle *vs* Apamin)	One-way ANOVA	0.0321
	E (Vehicle *vs* XE991)	One-way ANOVA	0.0051
	F (Healthy Vehicle *vs* $ALS^{C9orf72}$ Vehicle)	Two-way ANOVA	0.002
	F ($ALS^{C9orf72}$ Vehicle *vs* $ALS^{C9orf72}$ Apamin)	Two-way ANOVA	0.0052
	F ($ALS^{C9orf72}$ Vehicle *vs* $ALS^{C9orf72}$ XE991)	Two-way ANOVA	0.0230
EV 1	D	Welch`s t test	0.0425
	E	Mann-Whitney test	0.0286
EV 2	A	Welch`s t test	0.0195
	B	Welch`s t test	0.0444
	D (Healthy *vs* $ALS^{C9orf72}$, DIV 70)	Two-way ANOVA	0.005
EV 3	B	Mann-Whitney test	<0.001
	C	Mann-Whitney test	<0.001
	D	Welch`s t test	0.0029
	E	Welch`s t test	0.0038
EV 4	A	Mann-Whitney test	0.0111
	B	Mann-Whitney test	0.0085
EV 5	A (Vehicle *vs* Apamin)	One-way ANOVA	0.0420
	A (Vehicle *vs* XE991)	One-way ANOVA	0.0260
	B (Vehicle *vs* Apamin)	Kruskal-Wallis test	<0.001
	B (Vehicle *vs* Apamin)	Kruskal-Wallis test	<0.001
	C (Vehicle *vs* Apamin)	Kruskal-Wallis test	0.0756
	C (Vehicle *vs* XE991)	Kruskal-Wallis test	0.003
	D (Vehicle *vs* Apamin)	One-way ANOVA	0.0451
	D (Vehicle *vs* XE991)	One-way ANOVA	0.0395
	E (Vehicle *vs* Apamin)	Kruskal-Wallis test	<0.001
	E (Vehicle *vs* XE991)	Kruskal-Wallis test	<0.001
S3	A	Mann-Whitney test	<0.001
	B	Welch`s t test	0.0258
	C	Welch`s t test	0.0082
S4	C	Mann-Whitney test	0.004
	D	Welch`s t test	0.0244
	E	Mann-Whitney test	<0.001
S5	E	Mann-Whitney test	0.0245
	F	Welch`s t test	0.0392
	G	Welch`s t test	0.0031
S6	A	Mann-Whitney test	0.0107
	B	Welch`s t test	<0.001
	C	Welch`s t test	0.0293
	D	Mann-Whitney test	<0.001
	E	Fisher´s exact test	<0.001
	F	Mann-Whitney test	0.054
S8	B (Vehicle *vs* XE991 5µM)	Kruskal-Wallis test	<0.001

	B (Vehicle *vs* Apamin 500nM)	Kruskal-Wallis test	0.079
	B (Vehicle *vs* Charybdotoxin 100nM)	Kruskal-Wallis test	0.0173
	C (Vehicle *vs* Apamin)	One-way ANOVA	<0.001
	C (Vehicle *vs* XE991)	One-way ANOVA	<0.001
	D (Vehicle *vs* Apamin)	One-way ANOVA	0.0162
	D (Vehicle *vs* XE991)	One-way ANOVA	0.0036
	E (Vehicle *vs* Apamin)	One-way ANOVA	0.018
	E (Vehicle *vs* XE991)	One-way ANOVA	<0.001
	F (treatment effect)	Two-way ANOVA	0.0233
S9	Neurite length	Mann-Whitney test	<0.001
S10	(Vehicle *vs* Apamin)	Kruskal-Wallis test	<0.001
	(Vehicle *vs* XE991)	Kruskal-Wallis test	<0.001
S11	(Vehicle *vs* Apamin)	One-way ANOVA	0.0029
	(Vehicle *vs* XE991)	One-way ANOVA	0.037

Appendix Table S1. Exact p-values of the statistically significant comparisons presented within the manuscript.
