# Supplementary material for: Synaptic disruption and CREB‐regulated transcription are restored by K+ channel blockers in ALS
Source: EMBO Mol Med. 2021 Jun 14;13(7):e13131. doi: 10.15252/emmm.202013131 (PMC8261490; doi:10.15252/emmm.202013131)
Supplement: Supplementary file 2 — Expanded View Figures PDF [file EMMM-13-e13131-s004.pdf]

Expanded View Figures

Figure EV1. ALS^{C9orf72} MN display pathological accumulation of aggregates and autophagy blockade similar to ALS^{C9orf72} cells.

A List of the hiPSC lines used in this study.
B RNA foci were detected in confocal microscopy using a fluorescent GGGGCC probe in ALS^{C9orf72} neurons. Scale bar: 5 μm. Dashed line represents the cell soma.
C ALS^{C9orf72} MN accumulate aberrant perinuclear SQSTM1/p62^{+} aggresomes. Scale bar: 5 μm. Dashed line represents the cell soma.
D The levels of SQSTM1/p62 are significantly higher in ALS^{C9orf72} than in Healthy cells (Welch's *t*-test). $n = 3$ independent cultures for each hiPSC line. Scale bar: 10 μm.
E ALS^{C9orf72} MN display an autophagy blockade, as shown by the reduced levels LC3 II (Mann–Whitney test). $n = 4$ independent cultures, with the lines ALS^{C9orf72} I and Healthy I used as representative of the two genotypes.
F Representative TEM images showing cytosolic aggresomes in ALS^{C9orf72} (DIV 21) and ALS^{TBK1} (DIV 14) MN. Scale bar: 1 μm.
G Aberrant aggresomes in ALS MN are also enriched with the proteasome 20 s alpha subunit. Scale bar: 5 μm. Dashed line represents the cell soma.

Data information: $^{*}P < 0.05$. Error bars represent SEM. Arrows indicate the structures displayed at higher magnification. Exact *P*-values are reported in Appendix Table S1.
Source data are available online for this figure.

© 2021 The Authors

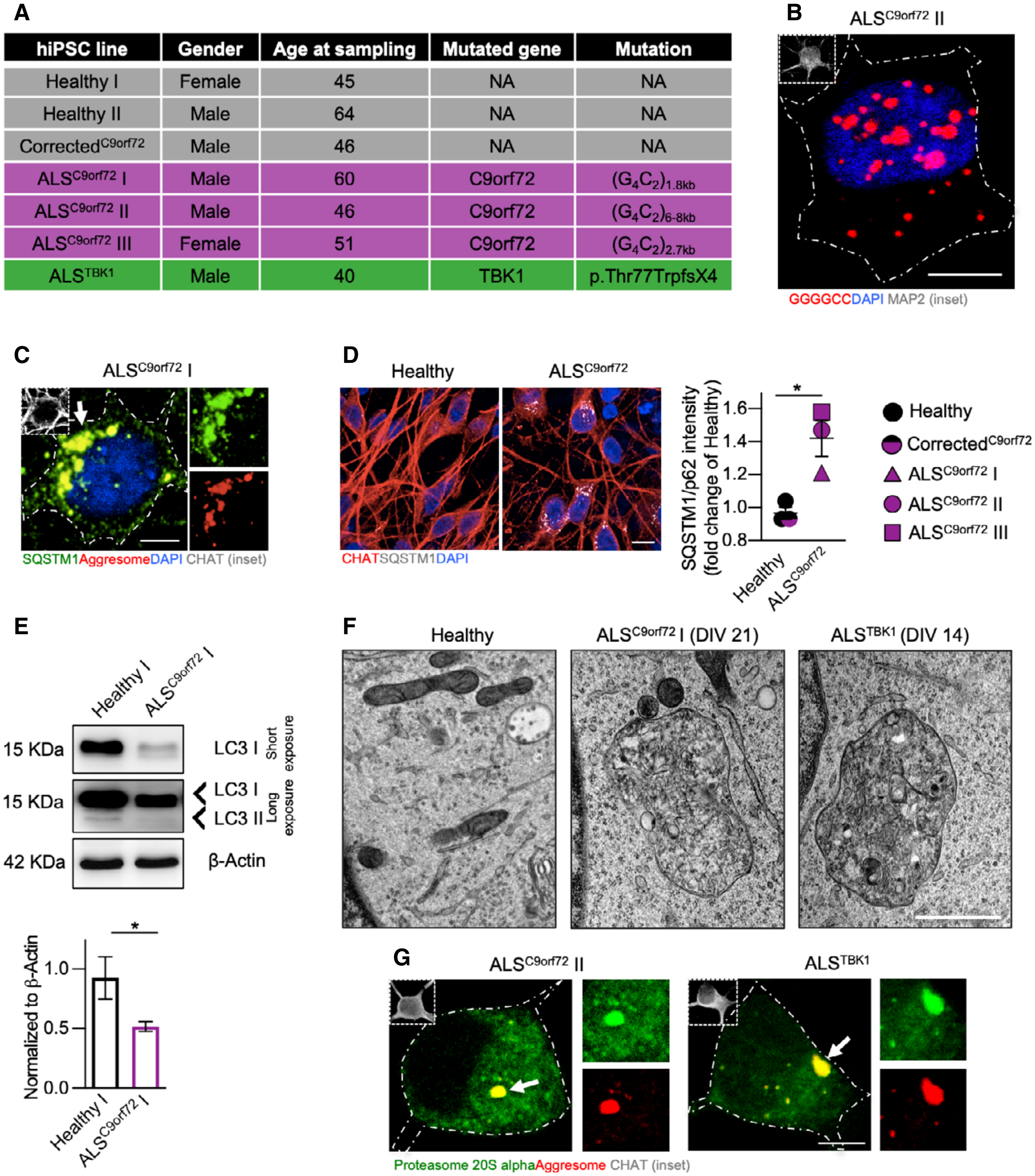

A

hiPSC line	Gender	Age at sampling	Mutated gene	Mutation
Healthy I	Female	45	NA	NA
Healthy II	Male	64	NA	NA
Corrected^{C9orf72}	Male	46	NA	NA
ALS^{C9orf72} I	Male	60	C9orf72	$(G_4C_2)_{1.8kb}$
ALS^{C9orf72} II	Male	46	C9orf72	$(G_4C_2)_{6\text{-}8kb}$
ALS^{C9orf72} III	Female	51	C9orf72	$(G_4C_2)_{2.7kb}$
ALS^{TBK1}	Male	40	TBK1	p.Thr77TrpfsX4

Figure EV1.

 © 2021 The Authors

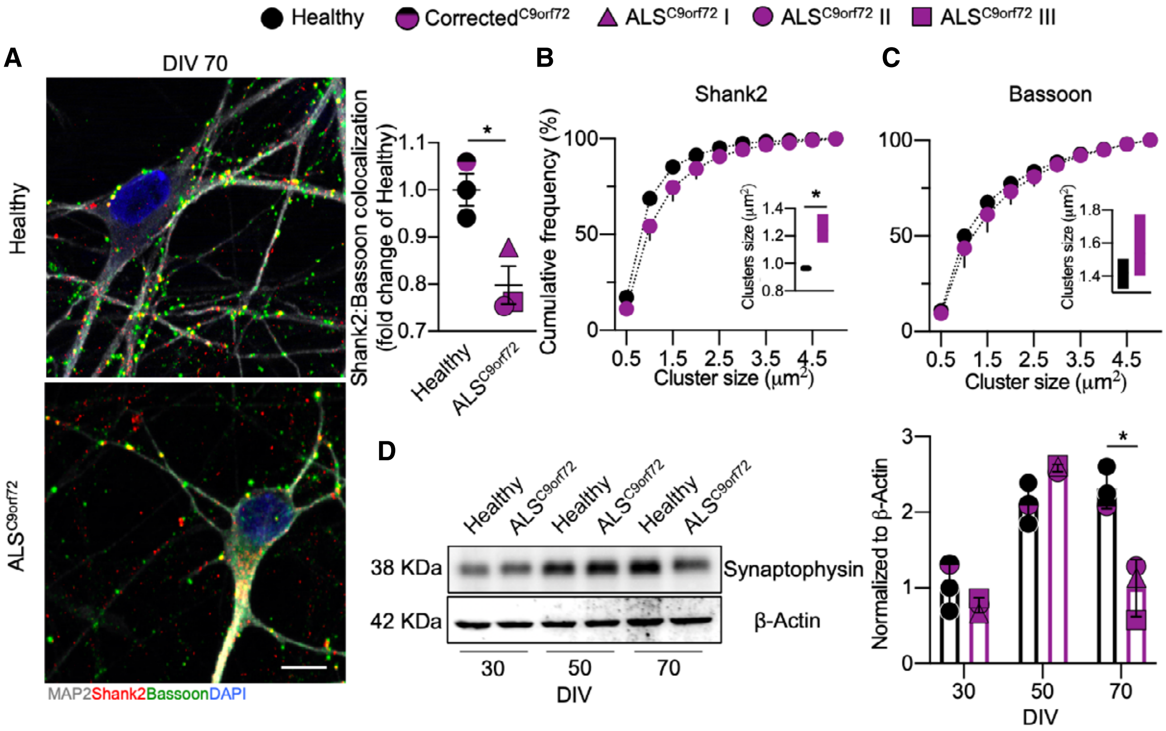

Figure EV2. Progressive loss of synapses in ALS.

A ALS MN have reduced Shank2:Bassoon synapses compared to Healthy controls (Welch's *t*-test). $n = 3$ independent cultures for each hiPSC line. Scale bar: 10 µm.

B, C Shank2^{+} dendritic clusters are significantly larger in ALS MN, while Bassoon puncta are comparable between genotypes (Welch's *t*-test). $n = 3$ independent cultures for each hiPSC line.

D Time course analysis of synaptophysin protein levels in human MN. $n = 3$ independent cultures for each hiPSC line (two-way ANOVA).

Data information: *$P < 0.05$. Error bars represent SEM. Exact *P*-values are reported in Appendix Table S1.

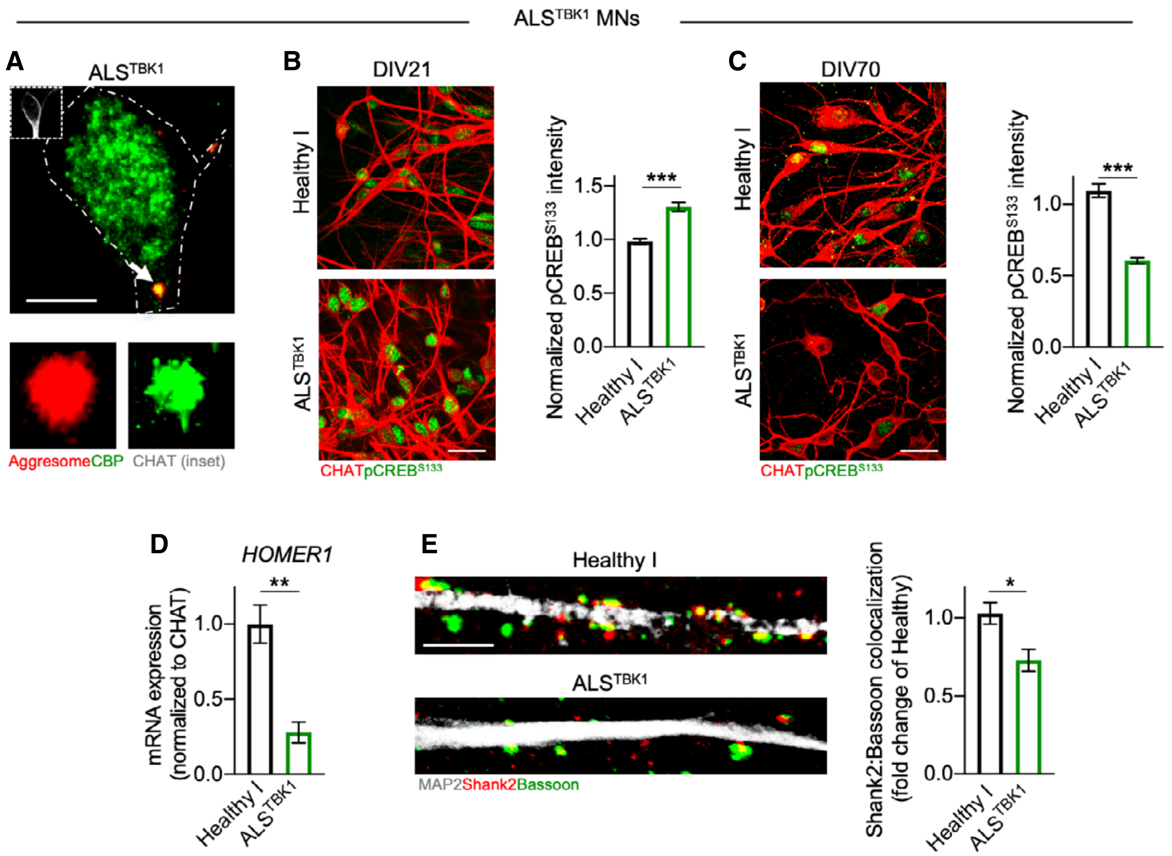

Figure EV3. ALS^{TBK1} MN are characterized by altered CREB activation and reduced synaptic contacts.

A CBP is sequestered within the aggresomes accumulating in ALS^{TBK1} MN. Scale bar: 5 μm. Dashed line represents the cell soma.

B DIV 21 ALS^{TBK1} MN have significantly higher nuclear levels of $pCREB^{S133}$ than an aged-matched Healthy control (Mann–Whitney test). $n = 3$ independent cultures. Scale bar: 25 μm.

C Similar to $ALS^{C9orf72}$ cultures, TBK1-mutant MN also show significantly lower phosphorylation of CREB than Healthy neurons at DIV 70 (Mann–Whitney test). $n = 3$ independent cultures. Scale bar: 25 μm.

D The expression of the CREB-dependent post-synaptic gene *HOMER1* is significantly lower in ALS^{TBK1} than Healthy I MN (Welch's *t*-test). $n = 3$ independent cultures.

E In agreement with the reduced activation of CREB, ALS^{TBK1} MN also show a significantly reduced number of excitatory synapses (Welch's *t*-test). $n = 3$ independent cultures. Scale bar: 5 μm.

Data information: $^{*}P < 0.05$; $^{**}P < 0.01$; and $^{***}P < 0.001$. Error bars represent SEM. Arrow indicates the structure displayed at higher magnification. Exact *P*-values are reported in Appendix Table S1.

© 2021 The Authors

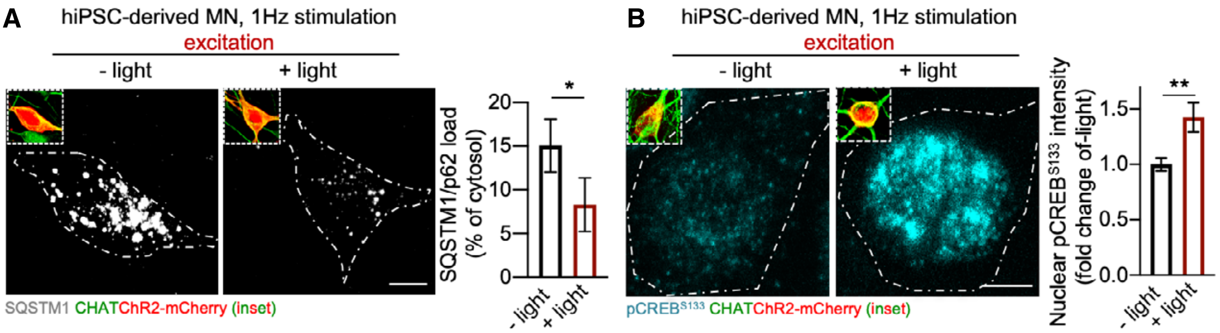

Figure EV4. Optogenetic enhancement of MN activity reduces the accumulation of SQSTM1 aggregates.

A 1 Hz optogenetic stimulation reduces the accumulation of aggregated SQSTM1/p62 in human $ALS^{C9orf72}$ MN (Welch's *t*-test). $n = 18$ MN analysed from three independent experiments. Scale bar: 10 µm. Dashed line represents the cell soma.

B The intensity of nuclear $pCREB^{S133}$ signal is significantly higher in stimulated $ALS^{C9orf72}$ MN than unstimulated ones. $n = 21$ MN analysed from three independent experiments (Welch's *t*-test). Scale bar: 5 µm. Dashed line represents the cell soma.

Data information: $*P < 0.05$ and $**P < 0.01$. Error bars represent SEM. Exact *P*-values are reported in Appendix Table S1.

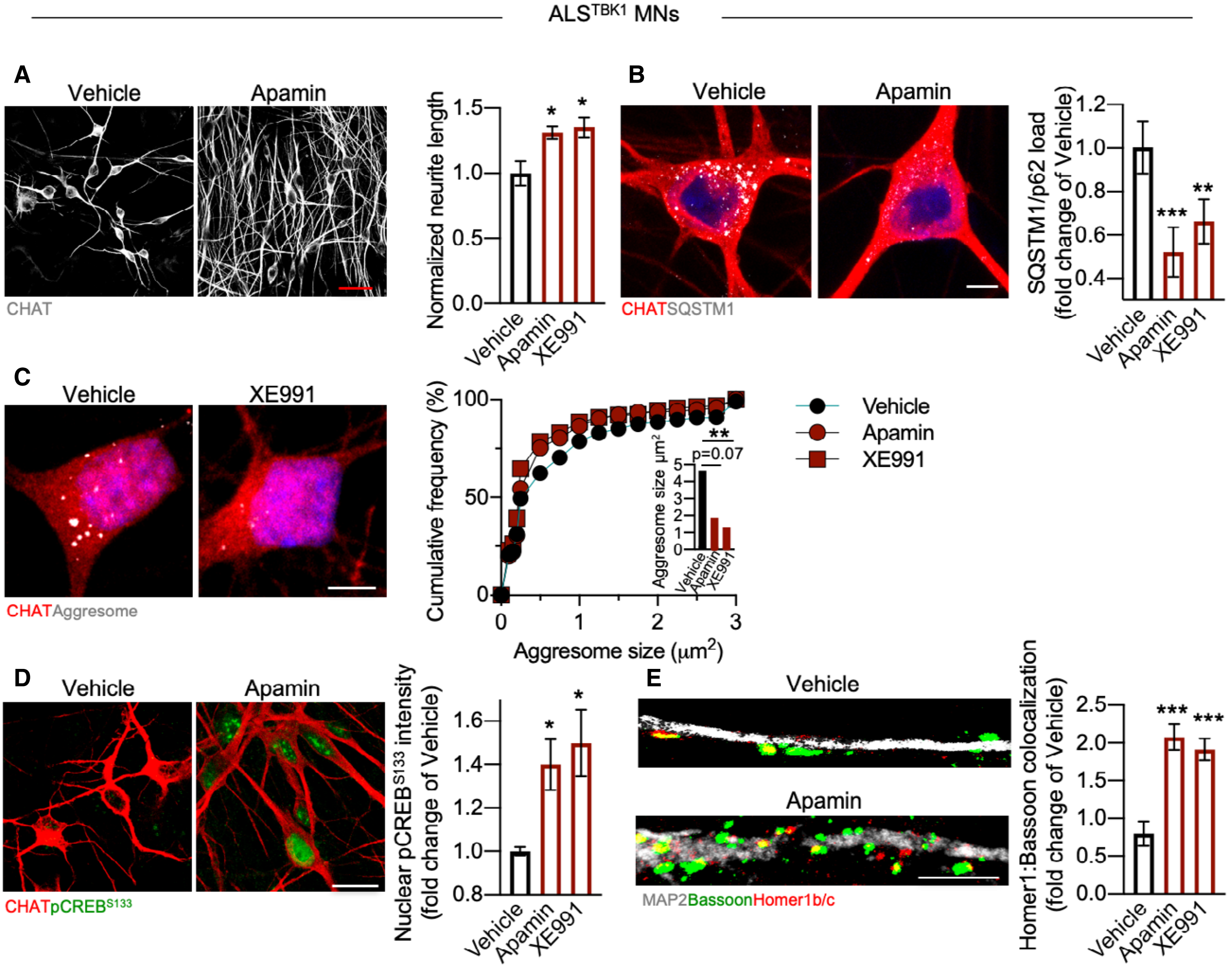

Figure EV5. Apamin and XE991 exert a neuroprotective effect also in ALS^{TBK1} MN.

A Apamin and XE991 increase the neurite length of ALS^{TBK1} MN (one-way ANOVA followed by Dunnett's multiple comparison test). $n = 3$ independent treatments. Scale bar: 50 μm.

B Both K^+ channel blockers reduce the accumulation of aggregated SQSTM1 in TBK1-mutant cells (one-way ANOVA followed by Dunnett's multiple comparison test). $n = 3$ independent treatments. Scale bar: 10 μm.

C K^+ channel blockade reduces also the size of cytotoxic aggresomes (Kruskal–Wallis test). $n = 3$ independent treatments. Scale bar: 5 μm.

D In Apamin- and XE991-treated cultures, the levels of phosphorylated CREB are significantly higher than in vehicle-treated ones (one-way ANOVA followed by Dunnett's multiple comparison test). $n = 3$ independent treatments. Scale bar: 10 μm.

E Apamin and XE991 rescue the loss of excitatory synapses also in TBK-mutant MN (Kruskal–Wallis test). $n = 3$ independent treatments. Scale bar: 5 μm.

Data information: $*P < 0.05$; $**P < 0.01$; and $***P < 0.001$. Error bars represent SEM. Exact P-values are reported in Appendix Table S1.

 © 2021 The Authors
