# Supplementary figures and images for: Synaptic disruption and CREB‐regulated transcription are restored by K+ channel blockers in ALS
Source: EMBO Mol Med. 2021 Jun 14;13(7):e13131. doi: 10.15252/emmm.202013131 (PMC8261490; doi:10.15252/emmm.202013131)

LC3A, short exposure

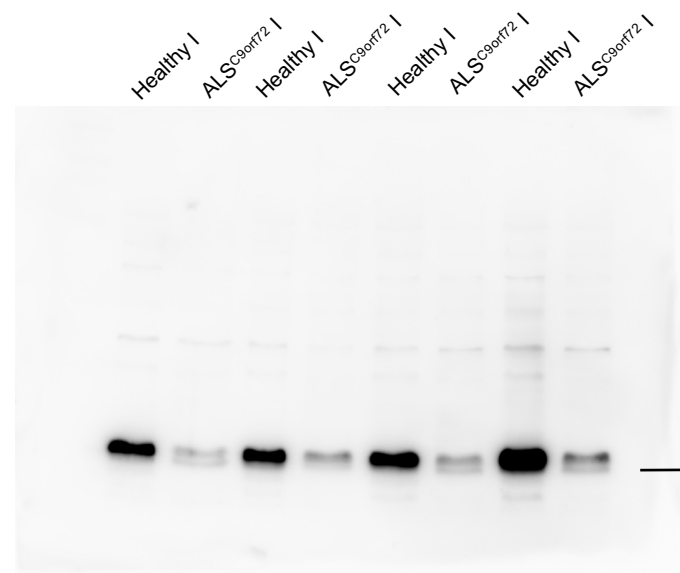

LC3A, long exposure

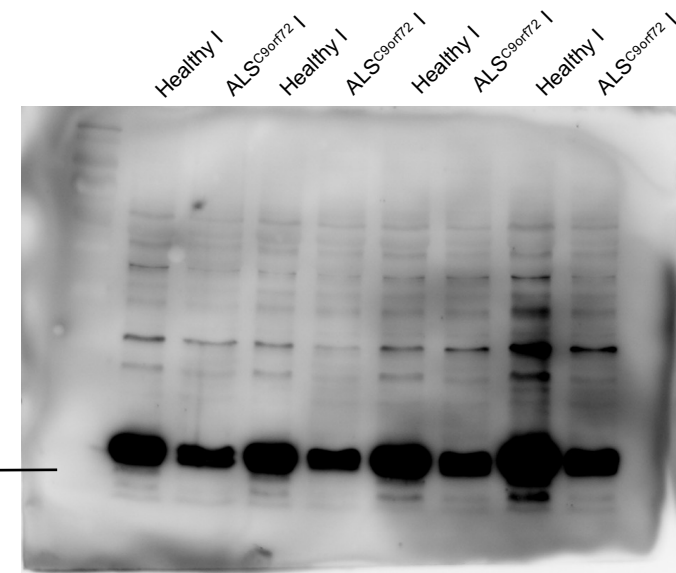

Actin  $\beta$

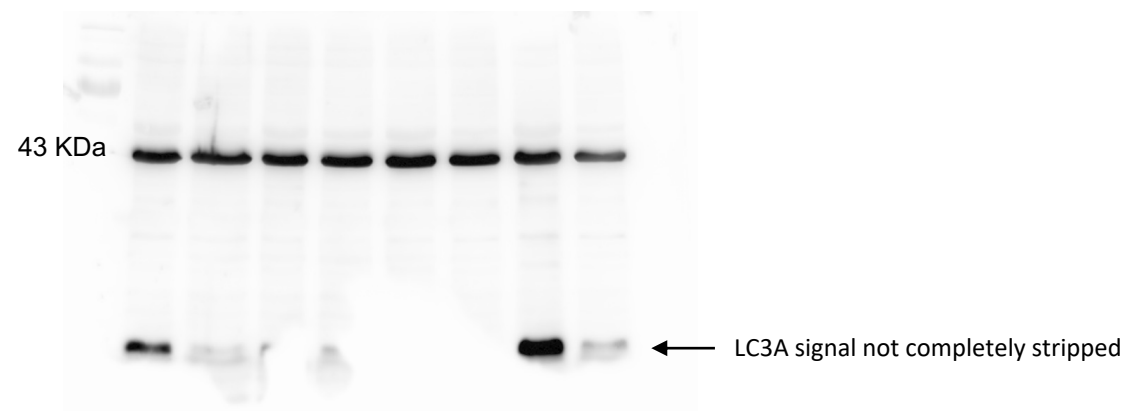

Supplement: Supplementary file 3 — Source Data for Expanded View [file EMMM-13-e13131-s001.zip › emmm202013131-sup-0003-SDataEV/emmm202013131-sup-0003-SDataFigEV1.pdf]
